# Quantification of Hepatitis E Virus in Naturally-Contaminated Pig Liver Products

**DOI:** 10.3389/fmicb.2016.01183

**Published:** 2016-08-03

**Authors:** Sandra Martin-Latil, Catherine Hennechart-Collette, Sabine Delannoy, Laurent Guillier, Patrick Fach, Sylvie Perelle

**Affiliations:** Université Paris Est (ANSES), Laboratory for Food SafetyMaisons-Alfort, France

**Keywords:** HEV, pig liver, quantification, RT-dPCR, RT-qPCR

## Abstract

Hepatitis E virus (HEV), the cause of self-limiting acute hepatitis in humans, is widespread and endemic in many parts of the world. The foodborne transmission of HEV has become of concern due to the identification of undercooked pork products as a risk factor for infection. Foodborne enteric viruses are conventionally processed by quantitative RT-PCR (RT-qPCR), which gives sensitive and quantitative detection results. Recently, digital PCR (dPCR) has been described as a novel approach to genome quantification with no need for a standard curve. The performance of microfluidic digital RT-PCR (RT-dPCR) was compared to RT-qPCR when detecting HEV in pig liver products. The sensitivity of the RT-dPCR assay was similar to that of RT-qPCR, and quantitative data obtained by both detection methods were not significantly different for almost all samples. This absolute quantification approach may be useful for standardizing quantification of HEV in food samples and may be extended to quantifying other human pathogens in food samples.

## Introduction

Hepatitis E virus (HEV) is a small non-enveloped single-stranded positive-sense RNA virus belonging to the Hepeviridae family (Emerson et al., 2004). HEV infection is generally asympto matic and is most commonly manifested as a self-limiting acute hepatitis in immunocompetent individuals (Aggarwal and Jameel, 2011).

The four genotypes able to infect humans have been recently classified into the Orthohepevirus A species within the Orthohepevirus genus (Smith et al., 2014). Genotypes 1 and 2 only infect humans, and are restricted to specific geographical areas (i.e., Asia, Africa, and Mexico). They often spread among the population as waterborne open epidemic outbreaks. On the contrary, genotypes 3 and 4 have also been isolated from different animal species and their zoonotic transmission is responsible for sporadic human cases, worldwide in the case of HEV genotype 3, and mainly in Asia for HEV genotype 4. Most episodes of zoonotic transmission associated with genotypes 3 and 4 are foodborne and have been linked to the ingestion of raw or undercooked meat, liver, and liver sausages from infected wild or domestic animals (boar, pigs, and deer) (Tei et al., 2003; Yazaki et al., 2003; Masuda et al., 2005; Deest et al., 2007; Colson et al., 2010). The presence of HEV in pig liver at grocery stores has been confirmed in the USA (Feagins et al., 2007), Japan (Okano et al., 2014), and various European countries, namely the Netherlands (Bouwknegt et al., 2007), the UK (Berto et al., 2012), Germany (Wenzel et al., 2011), Italy, Spain, and the Czech Republic (Di Bartolo et al., 2012). In France, foodstuffs containing raw pig liver were found to be contaminated by HEV (Colson et al., 2010; Martin-Latil et al., 2014).

A detection method developed to assess the risk related to the presence of HEV in food was found to give satisfactory performance, particularly in the framework of a French monitoring program (Martin-Latil et al., 2014). This method includes a virus concentration step by PEG followed by viral RNA extraction and subsequent detection by quantitative real-time RT-PCR (RT-qPCR).

To date, real-time RT-PCR has been one of the most promising detection methods due to its sensitivity, specificity and speed. The RT-qPCR assay has become the gold standard for quantitative viral diagnosis (Gunson et al., 2006). However, viral genome quantification is based on a standard curve which requires careful calibration and consistent source material. Therefore, due to differences in standard curve construction and potential analysis subjectivity, this relative quantitation approach has limitations, and may lead to inter-laboratory variations (Bustin and Nolan, 2004).

Digital PCR (dPCR) is a specific and sensitive endpoint absolute quantification approach that can determine target copy numbers without the need for a standard curve, thus leading to potentially more accurate and more precise quantification of nucleic acids. The principle of digital PCR relies on the partitioning of samples into multiple separate reactions that can be accomplished by generating micro-droplets or through the use of micro-fluidic chips. The signal in dPCR is measured after completing amplification, and the absolute number of target nucleic acid molecules in the sample is directly calculated from the ratio of positive to total partitions using binomial Poisson statistics (Dube et al., 2008; Pinheiro et al., 2012). This approach may also reduce the difficulty in quantifying viruses in the presence of inhibitors linked to matrix-type components analyzed in food or environmental virology (Racki et al., 2014).

In this study, a microfluidic digital RT-PCR chip with a 48-sample capacity was explored to determine the potential of this new concept of nucleic acid quantification in the field of food virology. The two most commonly process control viruses, Mengovirus, and murine norovirus (MNV-1) were used separately to check sample processing and an external amplification control (HEV transcribed RNA) to evaluate RT-PCR inhibition.

The reference RT-qPCR method was compared to the microfluidic digital RT-PCR (RT-dPCR) assay in terms of ability and sensitivity for detecting and quantifying HEV RNA genomes from naturally contaminated figatelli and pig liver sausages previously collected in the framework of a French surveillance plan.

## Materials and Methods

### Food Samples Containing Pig Liver

Food samples (figatelli and pig liver sausages) were collected within the framework of an official national surveillance plan organized by the French Ministry of Agriculture, Food and Forestry in 2011, according to EC Directive 2003/99/EC (2003) and EC Regulation 882/2004 (2004). Of the 400 samples qualitatively analyzed to detect HEV (Pavio et al., 2014), 70 were also quantitatively analyzed (Martin-Latil et al., 2014) and kept at -80°C. Six figatelli and six pig liver sausages previously found positive for the presence of HEV genomes were selected for this study according to their HEV contamination level. One figatellu and one pig liver sausage in which HEV genomes were not detected were used as negative samples.

### Process Control Viruses

The two most commonly used process controls, Mengovirus, or murine norovirus (MNV-1) were added before processing to monitor the whole viral extraction method. Dr. H. Virgin from Washington University (Saint Louis, MO, USA) supplied the ANSES Fougères Laboratory (Fougères, France) with the MNV-1 (CW1 strain), which was propagated in a mouse leukemic monocyte macrophage (RAW 264.7, ATCC TIB-71) cell line (Cannon et al., 2006). RAW 264.7 was grown at 37°C in an atmosphere containing 5% CO2 in DMEM supplemented with GlutaMAX^TM^, 1% non-essential amino acids and 10% fetal bovine serum (Life Technologies, Saint Aubin, France). The extracted RNA was quantified by measuring absorbance at 260/280 nm with a spectrophotometer (NanoDrop ND-1000) using the formula Copies = [weight (g) × 6.023 × 10^23^]/[size (bp) × 320.5], and its amplification was checked by RT-qPCR. Based on this approach, the production stock of MNV-1 had titres of approximately 1.36 × 10^12^ genome copies/mL.

A non-virulent mutant strain of mengovirus (vMC0 strain) (kindly provided by Albert Bosch, Department of Microbiology, Enteric Virus Group, University of Barcelona, Spain) was grown on HeLa cells (ATCC, CCL-2^TM^) as described by Costafreda et al. (2006). HeLa cells were grown at 37°C in an atmosphere containing 5% CO2 in Minimum Essential Media Glutamax^TM^ (MEM), 1% non-essential amino acids and 10% fetal bovine serum (Life Technologies). The extracted RNA was quantified by measuring absorbance at 260/280 nm with the NanoDrop ND-1000 using the formula Copies = [weight (g) × 6.023 × 10^23^]/[size (bp) × 320.5], and its amplification was checked by RT-qPCR. Based on this approach, the production stock of mengovirus had titres of approximately 6.68 × 10^11^ copies/mL.

### External Control (EC) RNA

An *in vitro* RNA transcript of HEV was used as an external control (EC) to monitor RT-PCR inhibition in RNA extracts.

The HEV cDNA corresponding to positions 5301-5371 of the genomic sequence (AB097812) was cloned in pGEM-T Easy Vector (Promega, Charbonnières-les-Bains, France) and propagated in *E. coli* One Shot^®^ TOP10F’ (Invitrogen, Cergy Pontoise, France). High-quality plasmid DNA containing this HEV region was purified using a QIAGEN Plasmid Midi kit (Qiagen, Courtaboeuf, France) according to the manufacturer’s protocol. The plasmid DNA was then digested with *SpeI* (Invitrogen) and transcripts were obtained using the MEGAscript^®^ kit (Ambion, Fisher Scientific, Illkirch, France) according to the manufacturer’s protocol. Synthesized RNA was treated once with RNase-Free DNase according to the manufacturer’s protocol to remove the DNA template following transcription, and purified using the RNeasy Mini kit (Qiagen). The synthesized RNA was confirmed with RT-qPCR and quantified by measuring absorbance at 260/280 nm with the NanoDrop ND-1000 (Thermoscientific, Courtaboeuf, France) using the formula Copies = [weight (g) × 6.023 × 10^23^]/[size (bp) × 320.5]. Aliquots of 20 μL with 10^8^ genome copies/μl were kept frozen at -80°C for later use as external amplification controls (EAC). One microlitre of EC RNA was added to an aliquot of RNA extract and tested using RT-qPCR. By comparing this result with the result of the EC RNA in the absence of an RNA extract, it is possible to determine the level of RT-PCR inhibition in each sample under test.

### Viral RNA Used as RNA Standards for HEV Quantification by RT-qPCR

Clarified HEV genotype 3f suspension was obtained from fecal samples of infected swine provided by ANSES’s Maisons-Alfort Laboratory for Animal Health. Pig HEV contaminated stools were obtained at Anses (Ploufragan) according to the animal welfare experimentation agreement (registration number C-22-745-1). The partial sequence was previously deposited with GenBank accession number JF718793.

The fecal sample was suspended in 10 mM Phosphate Buffered Saline of pH 7.4 to obtain a final 10% suspension (w/v), and then vortexed and centrifuged at 4000 *g* for 20 min at 4°C. The clarified fecal suspension was processed by the NucliSens^®^ easyMAG^TM^ Platform for viral genome extraction. The genomic titre was determined by RT-qPCR using an RT-qPCR standard curve obtained with the 10-fold diluted *in vitro* RNA transcripts. Viral RNA stock had a titre of approximately 1.75 × 10^6^ genome copies /mL. Aliquots were stored at -80°C for later use as RNA standards for HEV quantification by RT-qPCR.

### Sample Processing for Virus Recovery and Viral RNA Extraction

All the food samples were separated into 3 g portions and placed in a 400 mL polypropylene bag containing a filter compartment. To control losses of target virus which can occur at several stages during food sample analysis, a defined amount of process control virus—either 1.36 × 10^10^ genome copies of MNV-1 or 6.68 × 10^6^ genome copies of mengovirus—was inoculated on food samples (figatelli and pig liver sausages) (Martin-Latil et al., 2014; Hennechart-Collette et al., 2015). The inoculum was 100 μL of a dilution in diethylpyrocarbonate (DEPC)-treated water (Life Technologies) of the MNV-1 or mengovirus stock suspension. This was the earliest opportunity prior to virus extraction to check extraction efficiency. Uninoculated samples were used as a negative control for the process control virus.

Each food sample (3 g) was homogenized in 30 mL of distilled water using a Stomacher apparatus (Fisher Bioblock Scientific, Illkirch, France) at a normal velocity for 2 min. After an incubation of 10 min at room temperature with constant shaking, the filtrate was transferred to a 50-mL centrifuge tube and centrifuged at 8,000*g* for 15 min at 4°C to be clarified (removal of particulate debris). The decanted supernatant was supplemented with 10% (wt/vol) polyethylene glycol (PEG) 6000 (Sigma-Aldrich, Saint-Quentin Fallavier, France) and 0.3 M NaCl, and was then incubated for 2 h at 4°C. Viruses were concentrated by centrifugation of the solution at 8,000*g* for 30 min at 4°C. The supernatant was discarded and an additional centrifugation was performed at 8,000*g* for 5 min at 4°C to compact the pellet. This viral pellet was resuspended with 3 mL NucliSens^®^ easyMAG^TM^ lysis buffer (BioMérieux, Marcy l’Etoile, France) and lysed viral particles were processed on the NucliSens^®^ easyMAG^TM^ Platform (BioMérieux) for total nucleic acid extraction by the “off-board Specific A” protocol according to the manufacturer’s instructions. Lastly, nucleic acids were eluted in 70 μL of elution buffer, aliquoted and stored at -80°C.

Each experiment set, from spiking to RNA extraction, comprised seven figatelli and seven pig liver sausages and was performed 3 times. The same RNA extract (undiluted and 10-fold diluted) was analyzed in duplicate with the RT-qPCR assay and the digital RT-dPCR (RT-dPCR) to detect and quantify HEV genomes.

### Primers and Probes

The sequence of primer pairs and TaqMan probes used for HEV (viral genome and external control) and MNV-1 were those previously described (Martin-Latil et al., 2012; Martin-Latil et al., 2014). For HEV, the primers and TaqMan^®^ probe targeting the ORF2/ORF3 overlapping region were: 5′-CGGTGGTTTCTGGGGTGAC-3′ for the sense primer (HEV-5260-F), 5′-AGGGGTTGGTTGGATGAATATAG-3′ for the anti sense primer (HEV-5330-R) and 5′-ROX-GGGTTGATTCTCA GCCCTTCGC – BHQ2-3′ for the TaqMan probe (HEV-5280-T). For MNV, the sense primer (MNV-3193-F) was 5′-CCGCCATGGTCCTGGAGAATG-3′, the antisense primer (MNV-3308-R) was 5′-GCACAACGGCACTACCAATCTTG-3′ and the TaqMan probe (MNV-3227-T) was 5′-FAM–CGTCGTCGCCTCGGTCCTTGTCAA-BHQ1-3′.

For mengovirus, the sense primer (Mengo 110) was 5′-GCGGGTCCTGCCGAAAGT-3′, the antisense primer (Mengo 209) was 5′-GAAGTAACATATAGACAGACGCACAC-3′ and the TaqMan probe (Mengo 147) was 5′-FAM- ATCACATTAC TGGCCGAAGC-BHQ1-3′ (Costafreda et al., 2006).

All the primers and probes were purchased from Eurofins (Les Ulis, France).

### RT-qPCR Conditions on Bio-Rad’s CFX96

One-step RT-qPCR amplifications were performed on a CFX96^TM^ real-time PCR detection system from Bio-Rad (Marnes-la-Coquette, France). Reactions were performed in a 25 μL reaction mixture containing 1X RNA UltraSense^TM^ master mix and 1.25 μL of RNA Ultrasense^TM^ enzyme mix, which are components of the RNA UltraSense^TM^ One-Step Quantitative RT-PCR System (Life Technologies), 2 U RNAse inhibitor (Life Technologies), 5 μg of bovine serum albumin (Life Technologies), 600 nM (HEV-5260-F or MNV-1) or 500 nM (mengovirus) of forward primer, 600 nM (HEV-5330-R or MNV-1) or 900 nM (mengovirus) of reverse primer, 250 nM of probe (HEV-5280-T, MNV-1 or mengovirus) and 5 μL of RNA extract. Pure and 10-fold diluted RNA extracts obtained from each food sample were tested in duplicate. They were tested with and without the addition of 1 μL of 1.0 × 10^8^ genome copies of EAC (synthesized HEV RNA). Positive controls containing RNA extracted from virus suspensions and a negative control containing all the reagents except the RNA template were included in each run.

The one-step RT-qPCR program was 60 min reverse transcription of RNA at 55°C, followed by a 5 min denaturation step at 95°C, and lastly 40 cycles of 15 s at 95°C, 1 min at 60°C and 1 min at 65°C. The fluorescence was recorded by the apparatus at the end of the elongation steps (1 min at 65°C) for each amplification cycle. All samples were characterized by a corresponding cycle threshold (Ct) value. Negative samples gave no Ct value. A standard curve for each target (HEV, MNV-1, and mengovirus) was generated from 10-fold dilutions in ultrapure water of the titrated clarified suspension stocks. The slopes (*S*) of the regression lines were used to calculate the amplification efficiency (*E*) of the RT-qPCR reactions according to the formula *E* = 10^∣-1/^*^s^*^∣^-1.

### Quality Controls

Both the quality controls used to check the end-to-end viral extraction procedure and the presence of PCR inhibitors in RNA extracts, namely process control viruses and EAC, respectively, were analyzed using quantitative data obtained by RT-qPCR.

Extraction yields obtained for MNV-1 and mengovirus used as process controls were calculated with the following formula:

Number of viral genomes detected in 5 μL of undiluted RNA extract from tested samples/number of viral genomes detected in 5 μL of RNA extract from viral inoculum spiked on food samples X 100” (if 10-fold diluted RNA samples are used, multiply by 10 to correct for the dilution factor).

The recovery rates of the EAC were calculated with the following formula:

Quantity of EAC detected in 5 μL of RNA extracts from samples (undiluted or 10-fold diluted) / quantity of EAC detected in 5 μL of ultrapure water X 100.

### RT-dPCR Conditions on Fluidigm’s BioMark System

RT-dPCR amplifications were performed on a Fluidigm BioMark System using qdPCR 37K IFC digital array microfluidic chips (South San Francisco, CA, USA). With its nanoscale valves and channels, the BioMark Integrated Fluidic Circuit (IFC) controller partitions each of the 48 samples premixed with PCR reagents into a panel of 770 PCR reaction chambers (i.e., 36,960 individual qPCR reactions on a digital array). By counting the number of positive reactions, the number of target molecules in each sample can be accurately estimated according to Poisson distribution.

Reactions were performed in a 10 μL reaction mixture containing 1× of RNA UltraSense^TM^ master mix, 1× ROX reference dye and 0.44 μL of RNA UltraSense^TM^ enzyme mix, which are components of the RNA UltraSense^TM^One-step Quantitative RT-PCR System (Life Technologies), 1× of 20× GE Sample Loading Reagent (BioMark), 2 U RNAse inhibitor (Life Technologies), 600 nM of HEV forward primer (HEV-5260-F), 600 nM of HEV reverse primer (HEV-5330-R), 250 nM of HEV probe (HEV-5280-T) and 5.8 μL of RNA extract. A positive control containing HEV RNA extracted from viral stocks, and a negative control containing all the reagents except the RNA template, were included in each run. Pure and 10-fold diluted RNA extracts obtained from each sample were tested in duplicate. Six out of the 10 μL of reaction mix were loaded onto the chip with the IFC controller MX, but 0.65 μL was effectively partitioned into the 770 chambers of one panel, including 0.38 μL of RNA extract.

The temperature–time program was the same as that described above for RT-qPCR: 1 h at 55°C for the RT reaction, 5 min at 95°C as a hot start, and 45 cycles of 15 s at 95°C for denaturation, 1 min at 60°C for annealing, and 1 min at 65°C for extension.

### Statistical Analysis

All statistical analyses were performed with Statgraphics Centurion XV.II software.

One-way analysis of variance (ANOVA) was applied on EAC recovery rates to test (1) the effect of the type of food matrix (figatelli vs. pig liver sausages) and (2) the inter-assay variability among each of the food matrices.

The extraction yields obtained for MNV-1 were compared to those obtained for mengovirus through a one-way ANOVA according to the food matrix.

The influence of sub-sampling on HEV quantification was assessed by a one-way ANOVA for each food sample (six figatelli and six pig liver sausage samples) according to the detection method used (RT-qPCR vs. RT-dPCR). Both detection methods were then compared for HEV quantification through a one-way ANOVA.

The result of the ANOVA is a p value associated with the hypothesis that the means (EAC recovery rates, extraction yields or HEV genome copies) of all groups were the same.

## Results

### HEV Detection by RT-qPCR and Quality Controls

The RT-qPCR assay was used to detect HEV in figatelli and pig liver sausages and to assess both quality controls (EAC and process control virus).

#### Monitoring for RT-qPCR Inhibition in RNA Extracts

**FIGURE 1:**
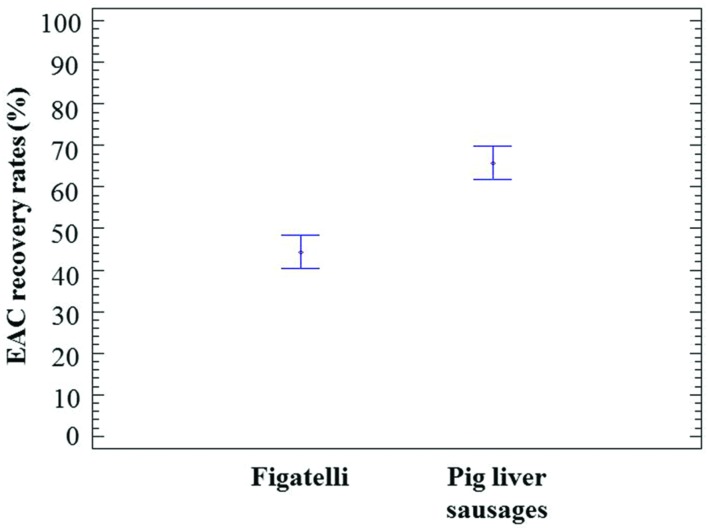
**Population marginal means with standard error of EAC recovery rates obtained in RNA extracts from figatelli and pig liver sausages**. Two means are significantly different if their intervals are disjoint and are not significantly different if their intervals overlap.

The EAC corresponding to the HEV RNA target was used to examine a potential inhibition of the HEV RT-qPCR assay in viral RNA extracts. The recovery rates of EAC in undiluted RNA extracts obtained from figatelli and pig liver sausages varied from 41.75 to 47.23% and from 56.44 to 84.73%, respectively (**Table 1**). These results showed that none of the samples showed significant evidence of inhibition. Nevertheless, statistical analysis showed that EAC recovery rates obtained in RNA extracts from pig liver sausages were significantly higher than those obtained in figatelli RNA extracts (ANOVA; *p*-value<10^-4^) (**Figure 1**), while they were not significantly different in samples belonging to the same food type (ANOVA; *P* = 0.9546 for figatelli; *P* = 0.3578 for pig liver sausages).

**Table 1:** Hepatitis E virus (HEV) detection in figatelli and pig liver sausages by RT-qPCR.

	HEV contami nation level	Sample names	EAC recovery rates (%)	Samples processed with MNV-1		Samples processed with mengovirus	
				MNV-1 recovery rates (%)	HEV positive subsamples	Mengovirus recovery rates (%)	HEV positive subsamples
**Figatelli**	**Low**	F1	42.29 ± 11.18	0.04 ± 0.02 (0/3)	3	1.40 ± 0.61 (2/3)	3
		F2	41.75 ± 7.630	1.34 ± 2.13 (1/3)	3	5.97 ± 3.38 (3/3)	3
	**Medium**	F3	45.94 ± 12.35	1.89 ± 3.08 (1/3)	3	4.69 ± 2.99 (3/3)	3
		F4	44.83 ± 10.40	1.31 ± 2.01 (1/3)	3	10.68 ± 6.19 (3/3)	3
	**High**	F5	47.23 ± 11.96	1.16 ± 1.83 (1/3)	3	4.16 ± 3.99 (2/3)	3
		F6	42.26 ± 9.030	0.15 ± 0.13 (0/3)	3	4.38 ± 2.07 (3/3)	3
	**Negative control**	F7	45.99 ± 10.89	7.14 ± 10.45 (3/3)	0	22.11 ± 27.72 (3/3)	0
	
	**Total subsamples with process control virus recovery rates >1%**	7		19	

**Pig Liver Sausages**	**Low**	S1	57.27 ± 22.75	1.12 ± 1.46 (1/3)	0	24.1 ± 25.34 (3/3)	0


		S2	58.57 ± 25.54	1.97 ± 1.98 (2/3)	1	16.12 ± 25.05 (2/3)	2
	**Medium**	S3	62.35 ± 24.66	1.74 ± 0.30 (2/3)	1	5.25 ± 8.43 (1/3)	3
		S4	68.81 ± 23.77	0.98 ± 1.38 (1/3)	3	24.65 ± 32.35 (3/3)	3
	**High**	S5	56.44 ± 26.82	3.75 ± 5.72 (1/3)	3	15.75 ± 0.37 (3/3)	3
		S6	72.61 ± 22.59	1.43 ± 1.29 (2/3)	3	15.88 ± 25.96 (2/3)	3
	**Negative control**	S7	84.73 ± 18.20	14.09 ± 9.73 (3/3)	0	25.50 ± 20.70 (3/3)	0
	
	**Total subsamples with process control virus recovery rates >1%**	12		17	

*One out of two Ct-positive HEV determinations in a single experiment set was considered a positive assay. The means of MNV-1 and mengovirus recovery rates ±SD were calculated with quantitative data obtained by RT-qPCR from undiluted RNA extracts. The number of experiments having process control recovery rates higher than 1% is indicated in brackets. The means of EAC recovery rate percentages ±SD were calculated for HEV using RT-qPCR from undiluted RNA extracts. For each sample, three experiments were performed with each process control virus, leading to six EAC recovery determinations. RNA extracts were tested twice*.

#### Monitoring for Viral Extraction Efficiency

To check the end-to-end viral extraction procedure, 1.36 × 10^10^ genome copies of MNV-1 or 6.68 × 10^6^ genome copies of mengovirus were added to the figatelli and pig liver sausage samples prior to their processing. The resulting extraction yields for MNV-1 and mengovirus from undiluted RNA extracts are reported in **Table 1**.

**FIGURE 2:**
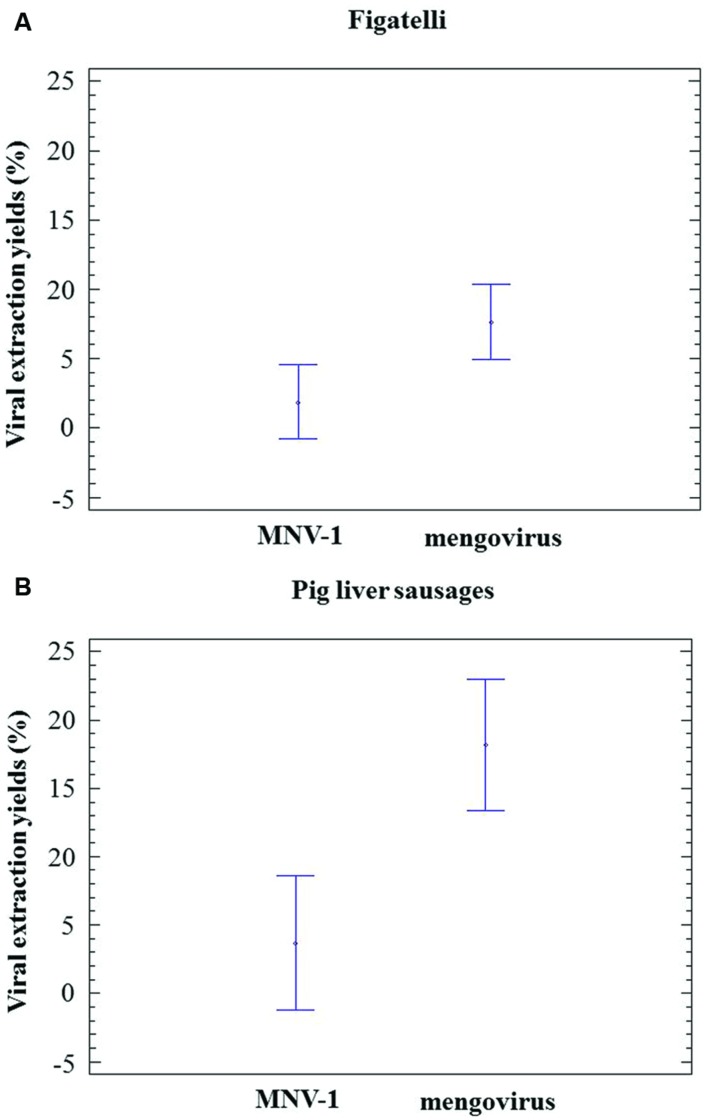
**Population marginal means with standard error of viral extraction yields (MNV-1 and mengovirus)**. **(A)** Figatelli. **(B)** Pig liver sausages. Two means are significantly different if their intervals are disjoint and are not significantly different if their intervals overlap.

Murine norovirus-1 and mengovirus were consistently detected in all RNA extracts obtained from figatelli and pig liver sausages. In figatelli, the recovery rates obtained for MNV-1 and mengovirus ranged from 0.04 to 7.14% and from 1.40 to 22.11%, respectively. Statistical analysis showed that they were significantly higher for mengovirus than for MNV-1 (ANOVA; *P*-value = 0.0351) (**Figure 2A**). In the same way, the recovery rates obtained for mengovirus (5.25-25.50%) in pig liver sausages were significantly higher than those for MNV-1 (0.98-14.09%) (ANOVA; *p*-value = 0.0042) (**Figure 2B**). The results showed that recovery rates obtained for process control viruses were higher than 1% for seven figatellu subsamples spiked with MNV-1 and 19 subsamples spiked with mengovirus. Nevertheless, all figatellu subsamples (F1 to F6) tested positive to the presence of HEV (in at least one of the two HEV RT-qPCR reactions). The recovery rates of MNV-1 and mengovirus were higher than 1% for 12 out of 21 and 17 out of 21 pig liver sausage subsamples, respectively. HEV was detected in three and four pig liver sausage samples spiked with MNV-1 (S4 to S6) and mengovirus (S3 to S6), respectively, for all the subsamples.

### Detection of HEV by RT-qPCR vs. RT-dPCR

#### HEV Quantification on Standard Materials Used for RT-qPCR Assays

Hepatitis E virus quantification was firstly assessed on serial dilutions of HEV RNA extracted from the clarified stool supernatants which were titrated by using OD and used as a standard for HEV quantification in food matrices by RT-qPCR. The RT-qPCR and RT-dPCR assays have a limit of detection of 13 HEV genome copies/μl (based on OD quantification).

The number of theoretical expected HEV genome copies within 0.38 μL of RNA effectively loaded in the digital plate was calculated from OD quantification. RT-dPCR detected from 21.0 to 37.4% of the expected input standard copy number (**Table 2**).

**Table 2:** Hepatitis E virus quantification by RT-dPCR on standard materials used for RT-qPCR assays.

HEV genome copies/μl (OD)	RT-qPCR HEV genome copies/assay (5 μl)	RT-dPCR Theoretical expected quantification (OD) HEV genome copies/assay (0.38 μl)	RT-dPCR Absolute quantification HEV genome copies/assay	% of quantification of expected input standard by RT-dPCR
250	1250 (3/3)0	95	35.5 ± 13.1 (3/3)	37.4
125	625 (3/3)	48	12.0 ± 6.3 (3/3)	25.0
25	125 (3/3)	10	3.0 ± 1.4 (3/3)	30.0
**13**	**065 (3/3)**	**5**	**1.0 ± 1.4 (3/3)**	**20.0**
3	015 (2/3)	1	0.3 ± 0.5 (1/3)	30.0

*The number of HEV genome copies (GC) of standard materials was determined by OD (spectrophotometer). The number of HEV GC per RT-qPCR assay and the theoretical expected HEV GC per RT-dPCR were calculated from OD by taking into account the volume of each assay (5 μl per RT-qPCR assay vs. 0.38 μl RT-dPCR per assay). The absolute quantification of HEV obtained by RT-dPCR was then compared to the theoretical expected HEV GC obtained by OD. The percentage of HEV quantification by RT-dPCR was calculated as follows: absolute quantification (RT-dPCR)/theoretical expected quantification (OD) X 100. All experiments were performed three times in duplicate. The number of positive assays is given in brackets and is shown in bold for the results corresponding to the limit of detection of the assay*.

As a whole, both detection methods have similar sensitivity and absolute quantification by RT-dPCR is 3- to 5-fold lower than quantification by RT-qPCR using standards quantified by OD.

#### HEV Detection in Naturally Contaminated Figatelli and Pig Liver Sausage Samples

Hepatitis E virus detection in figatelli and pig liver sausages by RT-qPCR and RT-dPCR was compared in terms of the numbers of HEV-positive reactions and HEV quantification (**Table 3**).

**Table 3:** Quantification of HEV in figatelli and pig liver sausages by RT-qPCR and RT-dPCR.

	HEV detection by RT-qPCR		HEV detection by RT-dPCR		HEV quantification RT-qPCR *vs* RT-dPCR	
Sample names	HEV-positive (out of 12)	HEV (Log_10_ genome copies)	HEV-positive	HEV (Log_10_ genome copies)	Mean (Log10 (HEV copies)_RT-qPCR_ – Log10 (HEV copies)_RT-dPCR_ per sub-sample)
F1	11	2.90 ± 0.86	10	2.81 ± 0.41	0.45
F2	12	3.17 ± 0.86	9	3.12 ± 0.78	0.36
F3	11	4.06 ± 0.72	10	3.89 ± 0.88	0.16
F4	12	4.34 ± 0.68	11	4.33 ± 1.07	0.08
F5	12	4.97 ± 0.98	11	4.88 ± 1.08	0.22
F6	12	4.73 ± 0.94	11	4.58 ± 1.31	0.21
S1	0	nd	1	2.26	/
S2	5	2.08 ± 0.71	6	2.31 ± 0.12	-0.11
S3	7	2.91 ± 0.95	8	2.77 ± 0.34	0.21
S4	11	3.62 ± 1.16	10	3.21 ± 0.69	0.56
S5	12	4.97 ± 0.25	12	4.34 ± 0.33	0.63
S6	10	6.32 ± 0.23	10	5.56 ± 0.11	0.76

*The results are presented as the mean of log_10_ (HEV genome copies) ±SD. For each sample, three experiments were performed with MNV-1 and three with mengovirus. Undiluted RNA extracts were tested twice, resulting in 12 cycle threshold (Ct) values for each sample. The number of positive Ct determinations is mentioned for HEV. HEV quantifications obtained by RT-qPCR and RT-dPCR are compared by calculating the differences for every subsamples and means of these differences are indicated for each sample*.

The difference between the detection methods (RT-qPCR vs. RT-dPCR) did not exceed one HEV-positive reaction except for one sample (F2). For five out of six samples of figatellu and one out of six samples of pig liver sausage, HEV was detected in only one more reaction with the RT-qPCR assay than with the RT-dPCR assay. Conversely, one more positive reaction for HEV was found by RT-dPCR for three out of six samples of pig liver sausage. The same number of HEV-positive reactions was found by both detection methods for two samples of pig liver sausage. As a whole, HEV could be detected by both methods at the same frequency.

To compare HEV quantification by both quantitative detection methods (RT-qPCR vs. RT-dPCR), the effect of sub-sampling was first evaluated through a one-way ANOVA for every figatellu and pig liver sausage according to the detection method used (RT-qPCR *versus* RT-dPCR).

For figatellu samples, the number of HEV genomes found by RT-qPCR and RT-dPCR varied significantly among subsamples (ANOVA; *p* < 0.05) except for sample F1 detected by RT-dPCR (ANOVA; *p* = 0.5217). For pig liver sausage samples, the number of HEV genomes found by RT-qPCR and RT-dPCR varied significantly among subsamples (ANOVA; *p* < 0.05) except for sample S2 detected by RT-qPCR (ANOVA; *p* = 0.4917). Therefore, RT-dPCR and RT-dPCR were compared by calculating the difference obtained for HEV quantification by both methods among subsamples and the mean of these differences are indicated for each sample in **Table 3**. The mean of differences in HEV quantification range from 0.08 to 0.45 for figatellu samples and from 0.11 to 0.76 for pig liver sausages. Moreover, the number of HEV genomes found by RT-qPCR was higher than that obtained by RT-dPCR except in the case of one pig liver sausage sample (S2). Nevertheless, the number of HEV genomes found by RT-dPCR was not significantly different from that obtained by RT-qPCR except in the case of two pig liver sausage samples (S5 (*p* < 0.0001); S6 (*p* < 0.0001)) as shown through a one-way ANOVA. Samples S5 and S6 had the highest differences in HEV quantification, with 0.63 and 0.76, respectively.

As a whole, the quantitative data obtained by both quantitative detection methods were not significantly different for almost all the samples.

## Discussion

The foodborne transmission of HEV is mainly due to consumption of raw or undercooked liver, meat or sausages from infected animal reservoirs such as pigs or wild boar (Yugo and Meng, 2013). However, there is currently no standardized method for detecting HEV in such products. The previously described method, based on the detection of HEV genomes by RT-qPCR (Martin-Latil et al., 2014), proved its effectiveness for HEV detection in naturally contaminated figatelli and pig liver sausages. Nevertheless, there is a growing interest in novel digital PCR technologies that allow precise and absolute quantification of nucleic acids, which could be useful for detecting viruses in food. In this study, a microfluidic-based digital RT-PCR (RT-dPCR) was compared to conventional RT-qPCR for detecting and quantifying HEV genomes in figatelli and pig liver sausages.

The RT-dPCR assay was comparable to RT-qPCR in terms of sensitivity, and HEV was detected in food samples at the same frequency by both detection methods. The reaction conditions (reaction mixtures and cycling protocol) were identical, but the RNA volume for RT-dPCR was 13-fold lower than for conventional RT-qPCR. Despite this smaller input volume for RT-dPCR, a similar sensitivity was observed in line with previous authors (Dhoubhadel et al., 2014; Coudray-Meunier et al., 2015).

The absolute quantification of HEV obtained by RT-dPCR for naturally contaminated figatelli and pig liver sausages was not significantly different from that obtained by RT-qPCR except for two pig liver sausages with the highest levels of HEV contamination. Indeed, the dilution of RNA samples may be necessary for high amounts to ensure that the number of target molecules per panel falls within an optimal range (Dong et al., 2015). The absolute quantification of HEV by RT-dPCR in food samples was always slightly lower than HEV quantification by RT-qPCR. An overestimation of the quantification of nucleic acids by RT-qPCR had already been reported and attributed to the methods used to determine the concentration of standards (e.g., OD determination) (Henrich et al., 2012; Sanders et al., 2013; Coudray-Meunier et al., 2015). Many studies highlight the problem of using standards for nucleic acid quantification by RT-qPCR since they themselves can be a source of error. Therefore, digital PCR is a promising new technology with the advantage of quantifying nucleic acid without the need of a standard curve. It could also be helpful in smoothing out variations in the quantitative data obtained by different laboratories (Brunetto et al., 2014; Hayden et al., 2015). Recently, it has also been used for rotavirus quantification in different types of surface water (Racki et al., 2014).

The RT-dPCR assay could also detect and quantify HEV genomes in any of the food types tested by taking into account sub-sampling. Indeed, sub-sampling appeared to be an influencing factor when quantifying HEV in figatelli and pig liver sausages by RT-qPCR and RT-dPCR. These results suggest that the distribution of HEV within naturally contaminated samples was heterogeneous and/or that virus extraction from food varied between subsamples. Since virus detection in food relies on a multi-step procedure, differences between repeat experiments have already been reported according to the pathogenic virus and food matrix (Hennechart-Collette et al., 2015). In food virology, the inclusion of quality controls is crucial for checking the whole virus extraction procedure. A standard method for detecting and quantifying noroviruses and hepatitis A virus in food was recently published by the European Committee for Standardisation (CEN) (group CEN/TC275/WG6/TAG4 “Detection of viruses in food”) (ISO/TS 15216-1, 2013; ISO/TS 15216-2, 2013). Despite the absence of a standardized method for detecting HEV in food, we took into account recommendations indicated in ISO/TS15216 to correctly interpret assay results for HEV detection in figatelli and pig liver sausages. Therefore, the described method for detecting HEV included the use of process controls to determine the level of acceptability of recovery efficiency throughout the whole process and the use of an EAC to examine RT-qPCR inhibition.

Mengovirus and MNV-1 were tested as process control viruses and the recovery rates found in the present study were comparable to those determined by other studies (Baert et al., 2011; Martin-Latil et al., 2014; Hennechart-Collette et al., 2015). Furthermore, recovery rates obtained for mengovirus were significantly higher than those obtained for MNV-1 regardless of the food matrix, as previously reported (Hennechart-Collette et al., 2015).

Based on the ISO/TS 15216 standard, samples were considered valid for analysis when recovery of the process control was higher than 1%. Our results showed that the processing of almost all samples (36 out of 42 subsamples) could be validated by using mengovirus, whereas only half of the subsamples were validated with MNV-1 (19 out of 42). Nevertheless, HEV could be recovered from samples having mengovirus or MNV-1 extraction yields lower than 1% except for one pig liver sausage.

The efficiency of EAC was over 40% for both matrices. By taking into account the fact that EAC recovery rates may be over 25% as indicated in the ISO/TS, our results showed that none of the samples revealed significant evidence of RT-PCR inhibition. Nevertheless, the effect of PCR inhibitors on HEV detection was clearly dependent on the food matrix, since EAC recovery rates differed significantly between figatelli and pig liver sausages. Other food matrices considered at risk for HEV and showing high levels of PCR inhibition should be tested to evaluate the robustness of digital PCR in the presence of inhibitors. Indeed, digital PCR may offer an advantage over qPCR when dealing with inhibition-prone samples because individual micro-reactions mitigate the impact of inhibitors, as previously described by both ourselves and others (Dingle et al., 2013; Morisset et al., 2013; Nixon et al., 2014; Racki et al., 2014; Coudray-Meunier et al., 2015). Moreover, the price per sample for viral quantification was estimated at half the cost with RT-dPCR rather than RT-qPCR.

## Conclusion

This study showed a novel application of microfluidic RT-dPCR and demonstrates its good potential for rapid, sensitive and accurate quantification of HEV genome in food matrices. As a result, RT-dPCR could be used as absolute quantification approach to routinely monitor enteric viruses in various food samples.

## Author Contributions

Performed experiments: SM-L and CH-C. Analyzed data: SM-L and CH-C. Statistical study: S-ML and LG. Conceived and designed experiments: All authors. Wrote the paper and approved the final manuscript: All authors.

## Conflict of Interest Statement

The authors declare that the research was conducted in the absence of any commercial or financial relationships that could be construed as a potential conflict of interest.
